# Supplementary material for: Scalable gradients enable Hamiltonian Monte Carlo sampling for phylodynamic inference under episodic birth-death-sampling models
Source: PLoS Comput Biol. 2024 Mar 29;20(3):e1011640. doi: 10.1371/journal.pcbi.1011640 (PMC11006205; doi:10.1371/journal.pcbi.1011640)
Supplement: S1 Text — (PDF) [file pcbi.1011640.s001.pdf]

Supplementary Material

1 Likelihood Derivation

1.1 Formulas for Likelihood Related Functions

$$A_k = \sqrt{\left(\lambda_k - \mu_k - \psi_k\right)^2 + 4\lambda_k \psi_k}, \tag{19}$$

$$B_k = \frac{\left(1 - 2\left(1 - \rho_k\right) p_{k-1}(t_{k-1})\right) \lambda_k + \mu_k + \psi_k}{A_k} \tag{20}$$

$$p_k(t) = \frac{\lambda_k + \mu_k + \psi_k - A_k \frac{e^{A_k(t-t_{k-1})}(1+B_k)-(1-B_k)}{e^{A_k(t-t_{i-1})}(1+B_k)+(1-B_k)}}{2\lambda_k} \tag{21}$$

$$q_k(t) = \frac{4e^{A_k(t-t_{k-1})}}{\left(e^{A_k(t-t_{k-1})}(1+B_k) + (1-B_k)\right)^2} \tag{22}$$

$$g_1 = e^{A_k(t-t_{k-1})} \cdot (1+B_k) + (1-B_k) \tag{23}$$

$$g_2 = A_k(1 - \frac{2(1-B_k)}{g_1}) \tag{24}$$

$$g_3 = 1 - 2\left(1 - \rho_k\right) P_{k-1}(t_{k-1}) \tag{25}$$

1.2 Implementation Algorithm:

Detailed algorithm for likelihood calculation is shown below based on the equations listed in Section 2.2 of the main text and from the section above.

Algorithm 1: Likelihood Calculation

Initialize: $p_0(t_0) = 1$
for $k = 0, \ldots, K-1$ **do**
    /* Intermediate quantities */
    Load the value of $p_k(t_k)$
    Calculate A_{k+1}, B_{k+1} via Equation (19), (20)
    for $j = 0, \ldots, m_{k+1} - 1$ **do**
        Calculate $q_{k+1}(s_{j+1})$ via Equation (22)
        if s_{j+1} *is a serial sampling event* **then**
            Calculate $p_{k+1}(s_{j+1})$ via Equation (21)
        end
        if $j < m_{k+1} - 1$ **then**
            Calculate $I_k(E_j)$ via Equation (2)
        end
    end
    Calculate and store $p_{k+1}(t_{k+1})$ via Equation (21)
end
/* Likelihood */
Calculate $\mathbb{P}[\mathcal{T} \mid \boldsymbol{\lambda}, \boldsymbol{\mu}, \boldsymbol{\psi}, \boldsymbol{\rho}, \boldsymbol{r}, \boldsymbol{t}]$ via Equation (1)

2 Gradient Derivation

2.1 For $\frac{\partial \log \mathbb{P}_k(j)}{\partial \theta_k}$:

$$
\begin{aligned}
\frac{\partial q_k(t)}{\partial \theta_k} = & \frac{8e^{A_k(t-t_{k-1})}((t-t_{k-1})\frac{\partial A_k}{\partial \theta_k}(\frac{1}{2}\cdot g_1 - e^{A_k(t-t_{k-1})}\cdot(1+B_k))}{g_1^3} \\
& - \frac{\frac{\partial B_k}{\partial \theta_k}(e^{A_k(t-t_{k-1})}-1))}{g_1^3}
\end{aligned} \tag{26}
$$

$$
\frac{\partial A_k}{\partial \theta_k} = \begin{cases} \frac{\lambda_k - \mu_k + \psi_k}{A_k}, & \text{If } \theta = \lambda \\ \frac{-\lambda_k + \mu_k + \psi_k}{A_k}, & \text{If } \theta = \mu \\ \frac{\lambda_k + \mu_k + \psi_k}{A_k}, & \text{If } \theta = \psi \\ 0, & \text{If } \theta = \rho \end{cases} \tag{27}
$$

$$
\frac{\partial B_k}{\partial \theta_k} = \begin{cases} \frac{2\lambda_k p_{k-1}(t_{k-1})}{A_k}, & \text{If } \theta = \rho \\ \frac{A_k \cdot g_3 - \frac{\partial A_k}{\partial \theta_k}\cdot(g_3 \cdot \lambda_k + \mu_k + \psi_k)}{A_k^2}, & \text{Otherwise} \end{cases}
$$

$$
\frac{\partial p_k(t)}{\partial \theta_k} = \begin{cases} \frac{1}{2\lambda_k^2}(-\mu_k - \psi_k - \lambda_k \frac{\partial g_2}{\partial \lambda_k} + g_2), & \text{If } \theta = \lambda \\ -\frac{A_k}{\lambda_k}\frac{\big((1-B_k)(e^{A_k(t-t_{k-1})}-1)+g_1\big)\frac{\partial B_k}{\partial \rho_k}}{g_1^2}, & \text{If } \theta = \rho \\ \frac{1}{2\theta_k}(1 - \frac{\partial g_2}{\partial \theta_k}), & \text{Otherwise} \end{cases} \tag{28}
$$

$$
\frac{\partial Q_k(s_{j+1}, s_j)}{\partial \theta_k} = \frac{1}{q_k(s_{j+1})}\frac{\partial q_k(s_{j+1})}{\partial \theta_k} - \frac{1}{q_k(s_j)}\frac{\partial q_k(s_j)}{\partial \theta_k} \tag{29}
$$

$$
\begin{aligned}
\frac{\partial g_2}{\partial \theta_k} = & \frac{dA_k}{d\theta_k} - \frac{2}{g_1^2}\cdot\Bigg(g_1\left\{\frac{dA_k}{d\theta_k}(1-B_k) - \frac{dB_k}{d\theta_k}\cdot A_k\right\} \\
& - \left(e^{A_k(t-t_{k-1})}\frac{\partial A_k}{\partial \theta_k}(1+B_k)\cdot(t-t_{k-1}) + (e^{A_k(t-t_{k-1})}-1)\frac{\partial B_k}{\partial \theta_k}\right)\cdot A_k(1-B_k)\Bigg)
\end{aligned} \tag{30}
$$

2.2 For $\frac{\partial \log \mathbb{P}_k(j)}{\partial \theta_{k-i}}$ (i is an integer smaller than k):

$$\frac{\partial q_k(t)}{\partial \theta_{k-i}} = -\frac{8e^{A_k(t-t_{k-1})}\frac{\partial B_k}{\partial \theta_{k-i}}(e^{A_k(t-t_{k-1})}-1)}{g_1^3} \tag{31}$$

$$\frac{\partial B_k}{\partial \theta_{k-i}} = \frac{\partial B_k}{\partial p_{k-1}\left(t_{k-1}\right)} \cdot \frac{\partial p_{k-1}\left(t_{k-1}\right)}{\partial \theta_{k-i}} = \frac{-2\left(1-\rho_k\right)\lambda_k}{A_k}\frac{\partial p_{k-1}\left(t_{k-1}\right)}{\partial \theta_{k-i}} \tag{32}$$

$$\frac{\partial p_k(t)}{\partial \theta_{k-i}} = -\frac{A_k}{\lambda_k}\frac{\left((1-B_k)(e^{A_k(t-t_{k-1})}-1)+g_1\right)\frac{\partial B_k}{\partial \theta_{k-i}}}{g_1^2} \tag{33}$$

$$\frac{\partial Q_k(s_{j+1}, s_j)}{\partial \theta_{k-i}} = \frac{1}{q_k\left(s_{j+1}\right)}\frac{\partial q_k\left(s_{j+1}\right)}{\partial \theta_{k-i}} - \frac{1}{q_k(s_j)}\frac{\partial q_k(s_j)}{\partial \theta_{k-i}} \tag{34}$$

2.3 Implementation Algorithm:

We implement a recursive algorithm to compute the necessary gradient of the log-likelihood within our rate parameter space. Intermediate quantities are stored in between epochs to alleviate computational burden. Detailed algorithm is shown below based on the equations listed in 2.5 and previous sections in the supplement.

Algorithm 2: Gradient Calculation

Initialize: $p_0(t_0) = 1$
for $k = 0, \dots, K-1$ **do**
  `/* Intermediate quantities */`
  if $k == 0$ **then**
    Calculate $\frac{\partial A_1}{\partial \theta_1}$, $\frac{\partial B_1}{\partial \theta_1}$ using $p_0(t_0)$ via Equation (27), (2.1)
  end
  else if $k \geq 1$ **then**
    Load the values of $\{\frac{\partial p_k(t_k)}{\partial \theta_i}\}_{i=1}^{k}$
    Calculate $\frac{\partial A_{k+1}}{\partial \theta_{k+1}}$, $\{\frac{\partial B_{k+1}}{\partial \theta_i}\}_{i=1}^{k+1}$ using $\{\frac{\partial p_k(t_k)}{\partial \theta_i}\}_{i=1}^{k}$ via Equation (27), (2.1), (32)
  end
  Calculate and store $\{\frac{\partial p_{k+1}(t_{k+1})}{\partial \theta_i}\}_{i=1}^{k+1}$ using $\{\frac{\partial B_{k+1}}{\partial \theta_i}\}_{i=1}^{k}$ via Equation (28), (33)
  `/* Gradient */`
  Calculate $\{\frac{\partial \log \mathbb{P}_k(j)}{\partial \theta_i}\}_{i=1}^{k}$ via Equations (11)-(18) in Section 2.5
end

3 Prior distributions for EBDS models

3.1 HIV dynamics in Odesa, Ukraine

We refer to the prior settings on the compound parameters from previous work [1], and try to roughly match their priors by adopting the following prior distributions on each of the rate parameters. Note that the sampling proportion was fixed to 0 before the first sampling date in their study, so we also set the sampling rate to 0 for the last two epochs for consistency.

Parameter	Prior	Role
λ	Lognormal (Mean = 0.85, SD = 1.0)	Birth rate
μ	Lognormal (Mean = −0.25, SD = 1.0)	Death rate
ψ	Lognormal (Mean = −9.0, SD = 0.50)	Serial sampling rate
t_{or}	Uniform (Lower = 19, Upper = 60)	Age of phylogeny

Table A: Prior specifications for the EBDS model in HIV virus analysis

3.2 Seasonal Influenza in New York State

We follow the same framework for setting the priors for the GMRF-based model as in Section 3.3. Similarly, the prior distribution for the constant death rate is acquired by estimating the credible range for the duration of the infectious period according to reports by Centers for Disease Control and Prevention [2], with 95% confidence intervals encompassing 6 to 11 days. Comprehensive information regarding the specific prior distributions is shown in the following table:

Parameter	Prior	Role
λ_1^*	Normal (Mean = 3.08, SD = 1.17)	Log-scale birth rate at present
μ_k^*	Normal (Mean = 3.82, SD = 0.16)	Log-scale death rate for all epochs
ψ_1^*	Normal (Mean = −0.77, SD = 1.17)	Log-scale sampling rate at present
t_{or}	Normal (Mean = 12.5, SD = 15.0)	Age of phylogeny
α	Fixed to 2.0	Exponent of the MRF
ϕ	Gamma (Shape = 1.0, Scale = 1.0)	Transformed global scale of the MRF
ν_k	Fixed to 1.0	Local scale of MRF

Table B: Prior specifications for the EBDS model in Influenza virus analysis

3.3 Ebola epidemic in West Africa

We assume a constant death rate, μ for this data set, and we employ an empirical Bayes approach proposed by Magee *et al.* (2020) to set the prior on the first log-birth-rate and log-sampling-rate in our Bayesian bridge MRF models [3]. The prior for the constant death rate is obtained from an estimation of the plausible duration of infectious period with 95% confidence intervals covering 8 to 40 days [4]. The detailed prior distributions can be found in the table below:

Parameter	Prior	Role
λ_1^*	Normal (Mean = 1.26, SD = 0.58)	Log-scale birth rate at present
μ_k^*	Normal (Mean = 3.02, SD = 0.41)	Log-scale death rate for all epochs
ψ_1^*	Normal (Mean = 1.27, SD = 0.58)	Log-scale sampling rate at present
t_{or}	Normal (Mean = 1.89, SD = 15.0)	Age of phylogeny
α	Fixed to 0.25	Exponent of the MRF
ϕ	Gamma (Shape = 1.0, Scale = 1.0)	Transformed global scale of the MRF
ν_k	Exponentially tilted stable distributions	Local scale of Bayesian bridge MRF
ξ	Fixed to 2.0	Slab width of Bayesian bridge MRF

Table C: Prior specifications for the EBDS model in Ebola virus analysis

4 Additional efficiency metrics

	Run time (hours)		Minimum ESS/state		
	MH-MCMC	HMC	MH-MCMC	HMC	HMC Speedup
HIV (10 epochs)	1.25×10^{1}	5.12×10^{-2}	8.00×10^{-7}	8.21×10^{-4}	1.03×10^{3}
Influenza (78 epochs)	8.23×10^{2}	1.04×10^{1}	2.35×10^{-8}	4.83×10^{-6}	2.06×10^{2}
Ebola (24 epochs)	3.77×10^{1}	2.97×10^{0}	1.22×10^{-6}	3.43×10^{-5}	2.81×10^{1}

Table D: Relative speedup in terms of effective sample size (ESS) per MCMC chain-state comparing HMC over MH-MCMC and required run time to reach a minimum ESS > 200 across all EBDS model rates for all three examples

5 Inferred trajectories for birth/death/sampling rates

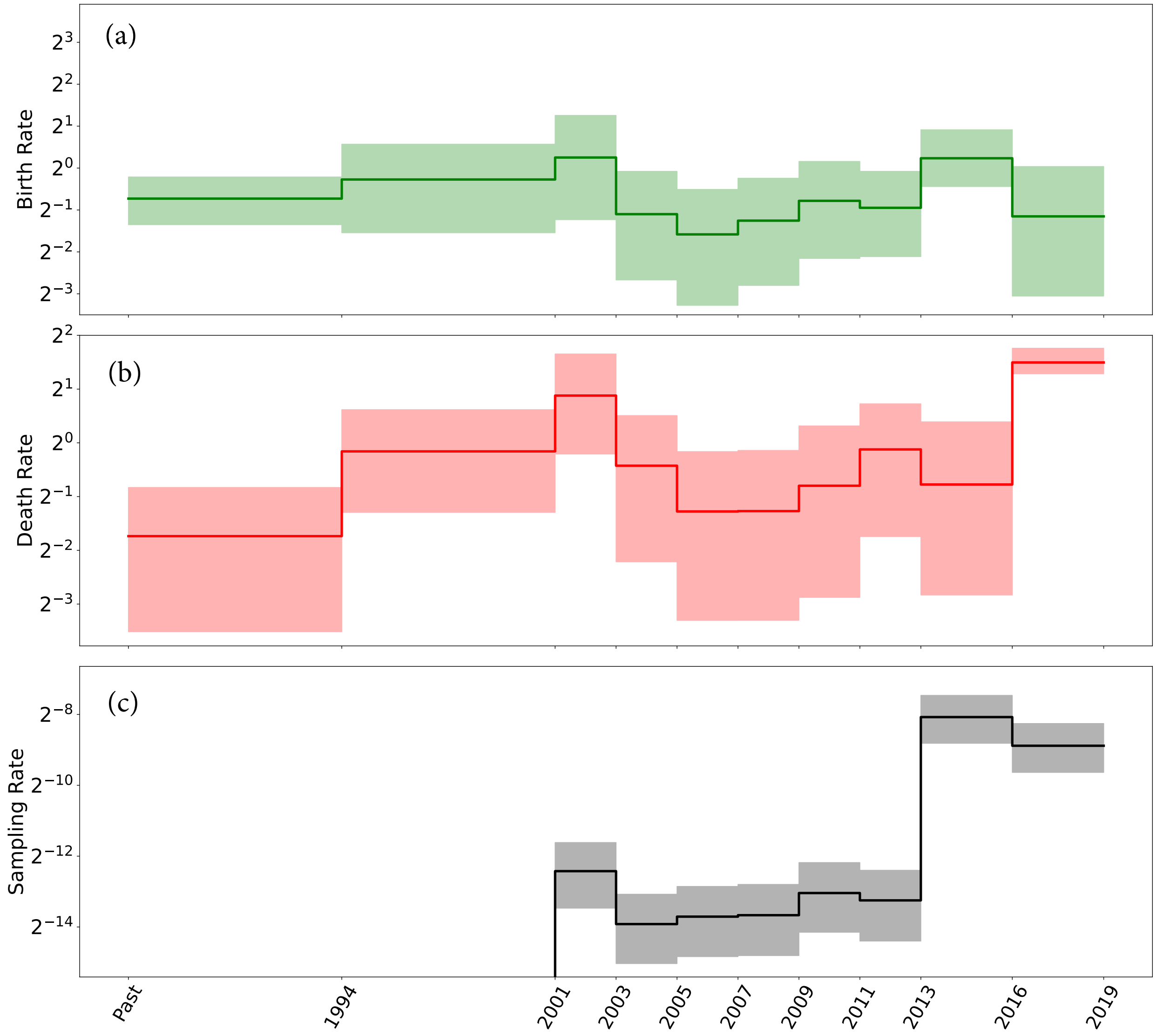

Figure A: HIV virus: Median (solid line) and 95% credible intervals indicated by the shaded areas of the (a) birth rate, (b) death rate, and (c) sampling rate estimates through time.

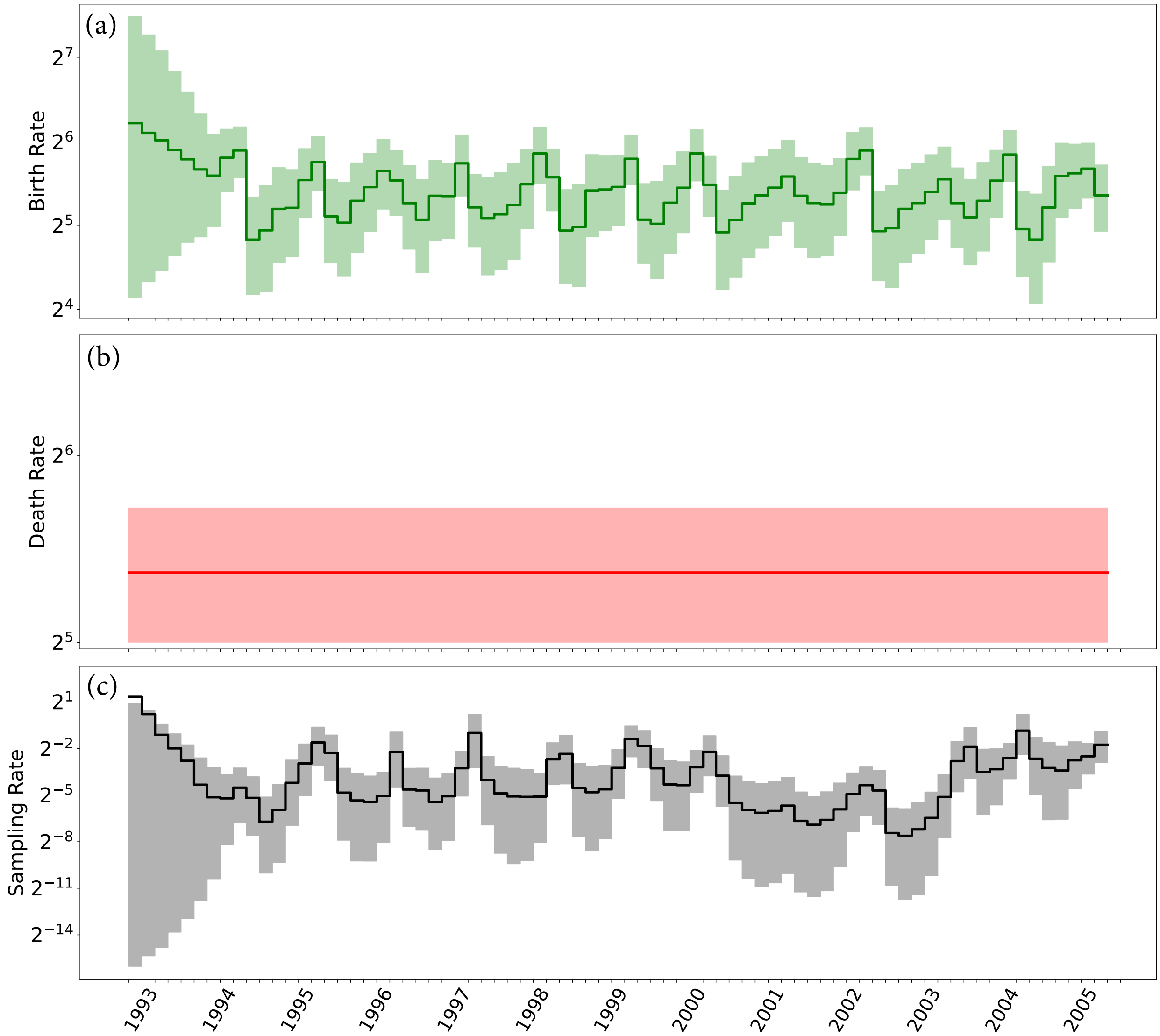

Figure B: Influenza virus: Median (solid line) and 95% credible intervals indicated by the shaded areas of the (a) birth rate, (b) death rate, and (c) sampling rate estimates through time.

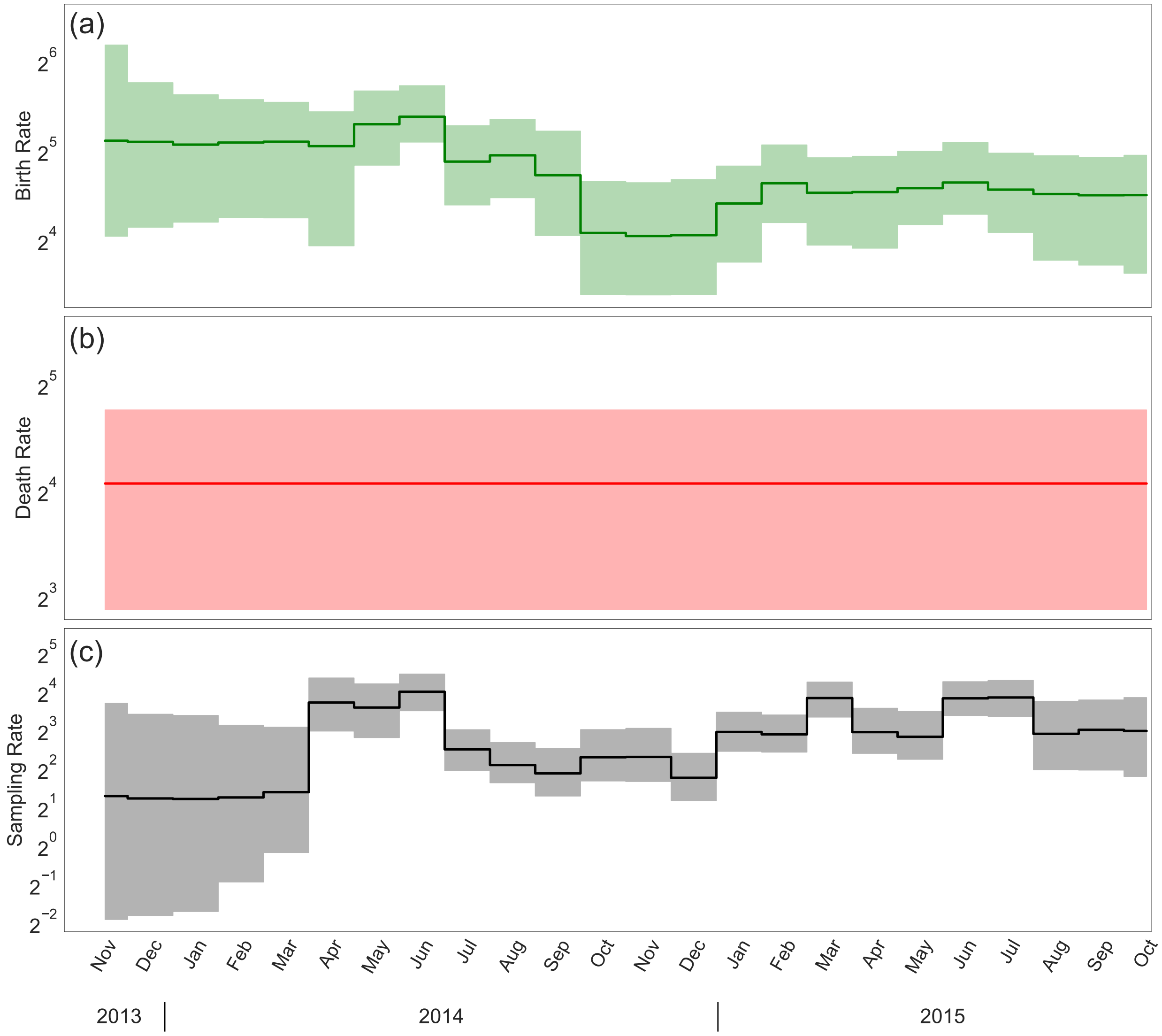

Figure C: Ebola virus: Median (solid line) and 95% credible intervals indicated by the shaded areas of the (a) birth rate, (b) death rate, and (c) sampling rate estimates through time.

6 Computational complexity of the nodewise likelihood

The computational complexity of evaluating node-based representations of the likelihood is much less explicit. First, we need to write out an equivalent expression for the likelihood of Equation 1 node-wise. It will be helpful to distinguish different types of samples. In particular, let us denote serially-sampled tips $\bar{\boldsymbol{u}}_\psi$ with a particular serially-sampled tip being $\bar{u}_{\psi i}$. With a slight abuse of notation, let us denote intensively-sampled tips $\bar{\boldsymbol{u}}_\rho$, with $\bar{\boldsymbol{u}}_{\rho i}$ denoting the *vector* of intensively-sampled tips at the ith intensive-sampling event. Then we can write

$$\begin{aligned}\mathbb{P}[\mathcal{T} \mid \boldsymbol{\lambda}, \boldsymbol{\mu}, \boldsymbol{\psi}, \boldsymbol{\rho}, \boldsymbol{r}, \boldsymbol{t}] = \log(q_{K(t_{or})}(t_{or})) + \left(\sum_{i=1}^{||\boldsymbol{v}||} \log(\lambda_{k(v_i)}) + \log(q_{k(v_i)}(v_i))\right) + \\ \left(\sum_{i=1}^{||\bar{\boldsymbol{u}}_\psi||} \log(\psi_{k(\bar{u}_{\psi i})}) + \log(r_{k(\bar{u}_{\psi i})} + (1 - r_{k(\bar{u}_{\psi i})})p_{k(\bar{u}_{\psi i})})) - \log(q_{k(\bar{u}_{\psi i})}(\bar{u}_{\psi i}))\right) + \\ \left(\sum_{i=1}^{K} ||\bar{\boldsymbol{u}}_{\rho i}|| \log(\rho_i) + (L(t_{i-1}) - ||\bar{\boldsymbol{u}}_{\rho i}||) \log((1 - \rho_i)q_{i-1}(t_{i-1})) + \right. \\ \left. ||\bar{\boldsymbol{u}}_{\rho i}|| \log(r_i + (1 - r_i)p_{i-1}(t_{i-1}))\right) \quad (35)\end{aligned}$$

The complexity here is not immediately apparent for a number of reasons. For one, the complexity appears to depend on the relative proportion of samples of different types, which affects the number of values of $p_k(t)$ and $q_k(t)$ which must be computed. Importantly, the complexity of computing those $p_k(t)$ and $q_k(t)$ is not immediately apparent either, and that these costs are somewhat hard to disentangle, as $p_k(t_i)$ builds recursively on $p_{k-1}(t_i)$ and $q_k(t)$ depends on $p_k(t)$.

6.1 Node lookups

Regardless of such ambiguities, all nodes in the tree require an interval lookup. For births, the lookup is required to find the correct λ_k term to use. For samples, the lookup is either to find

the appropriate sampling rate, for serial samples, or to determine to which intensive-sampling event a sample belongs, for intensive samples. The time requirement here depends on the algorithm, for a binary search it is $\mathcal{O}(\log(K))$, making the total lookup cost $\mathcal{O}(N\log(K))$.

6.2 How many computations of $q_k(t)$ are required?

In the worst, but most common, case, there are no intensive-sampling events and $q_k(t)$ must be computed for the times of all samples, all births, and all epoch times (note that even when ρ_i is 0, there is a term $L(t_i)\log(q_{i-1}(t_i))$ which must be computed in the final summation). In the best case, all samples are at intensive-sampling events, and $q_k(t)$ only needs to be computed for the times of all births and all epoch times. These are both $\mathcal{O}(N+K)$, though there is a factor of two's worth of variation in front of the N depending on which side of this spectrum a tree falls in. Calling the cost of computing $q_k(t)$ Q, this makes the contribution to the complexity here $\mathcal{O}(Q(N+K))$.

6.3 How many computations of $p_k(t)$ are required?

The likelihood contains a number of explicit computations of $p_k(t)$ in the terms pertaining to (both serially- and intensively-)sampled tips. When all samples are serial samples, there are $\mathcal{O}(N)$ direct computations of $p_k(t)$, while when all samples are intensive samples, there are $\mathcal{O}(K)$. Taking the cost of computing $p_k(t)$ to be P, the addition to the cost here is between $\mathcal{O}(PN)$ and $\mathcal{O}(PK)$.

6.4 What is the cost of computing $p_k(t)$ and $q_k(t)$?

We have thus far shown that the cost of computing the nodewise likelihood appears to be between $\mathcal{O}(N\log(K)+Q(N+K)+PN)$ and $\mathcal{O}(N\log(K)+Q(N+K)+PK)$. But this is not particularly revealing without considering P and Q.

While $q_k(t)$ depends on $p_{l:l<k}(t)$ through $\mathbf{A}$ and $\mathbf{B}$, once A_k and B_k have been computed,

let us assume (as we did when evaluating the cost of the interval-wise likelihood) that the cost of $q_k(t)$ is $\mathcal{O}(1)$. In other words, let us assume that $\mathcal{O}(Q(N+K)) = \mathcal{O}(P(N+K))$. This makes the implied cost of the nodewise likelihood between $\mathcal{O}(N\log(K) + P(N+K) + PN)$ and $\mathcal{O}(N\log(K) + P(N+K) + PK)$, which both simplify to $\mathcal{O}(N\log(K) + P(N+K))$. Naïvely, we might choose to compute $p_k(t)$ recursively every time we need it, which is $\mathcal{O}(K^2)$. In this case, the implied cost of the nodewise likelihood is $\mathcal{O}(N\log(K) + NK + K^2))$.

6.5 Precomputing $\mathbf{A}$ and $\mathbf{B}$

One can instead choose to pre-compute A_k, B_k, as once these are computed the cost to compute $p_k(t)$ and $q_k(t)$ becomes $\mathcal{O}(1)$. Working backwards from the present allows re-computation to be avoided. As we did when we approximated the cost of the interval-wise likelihood, we will take the cost of the update (computing (A_k, B_k) from (A_{k-1}, B_{k-1})) to be $\mathcal{O}(1)$. Thus, the cost of the precomputation is $\mathcal{O}(K)$. This puts the implied cost of computing the nodewise likelihood between $\mathcal{O}(N\log(K) + N + K)$.

6.6 Counting lineages at epoch times

Regardless of whether the model includes intensive-sampling (that is, regardless of whether $\boldsymbol{\rho} = 0$), one must compute $L(t_i)$ for all epoch times. This can be solved essentially the same way as the subintervals are obtained, at a cost of $\mathcal{O}(N + N\log(N))$. Alternately, it can be obtained by counting the number of births and sampled tips older (or younger) than each epoch time, at a cost of $\mathcal{O}(KN)$. This makes the lower end of the computational cost once again a range, from $\mathcal{O}(NK + N\log(K) + N + K)$ to $\mathcal{O}(N\log(K) + N\log(N) + N + K)$.

In practice, the constants in front of all the sorting and node-lookup terms appear to be so small as to be unnoticeable in real-world computation. We demonstrate this in our timing experiments in the next section. Thus, for all practical purposes, the likelihood appears to be $\mathcal{O}(N + K)$ regardless of representation, as long as one avoids recursive computation of $p_k(t)$.

7 Timing Experiments

With the reformulation of the likelihood and derivation of the analytical gradients, our method notably gains in speed, as we highlight in this section. For a comprehensive assessment, we compare our approach with four other specialized packages for EBDS model inference concerning likelihood calculations. These include the BDSKY [5] package within BEAST2 [6], TreePar [5] package in R [7] and RevBayes [8]. Furthermore, we present a benchmark comparing the gradient calculation efficiency of automatic differentiation implemented in VBSKY [9] package using JAX library [10] isolated from the variational inference procedure against our algorithm based analytical gradients implemented in BEAST.

To assess the scalability of the aforementioned methods in terms of likelihood/gradient calculation, we simulated a set of trees under the EBDS model with increasing number of tips. To investigate the scalability of different methods wrt the number of sequences, we fix the number of epochs to 5 for both likelihood and gradient calculation.

Regarding scalability with respect to the number of epochs, we adjust the model by progressively increasing the number of epochs. To keep other variables constant, we maintain the tree topology and set the number of tips at 12 (in scenarios where $K >> N$, this allows us to negate the effect of N in $\mathcal{O}(N+K)$) for likelihood computation. For gradient calculations, we set the number of tips to 8198 (to minimize the impact of K^2 in $\mathcal{O}(NK+K^2)$).

For methods that employ just-in-time (JIT) compilation, including BEAST, BEAST2 and VBSKY, we run a short MCMC chain or variational inference algorithm to compute likelihood or gradient across 100,000 iterations and take the average run time.

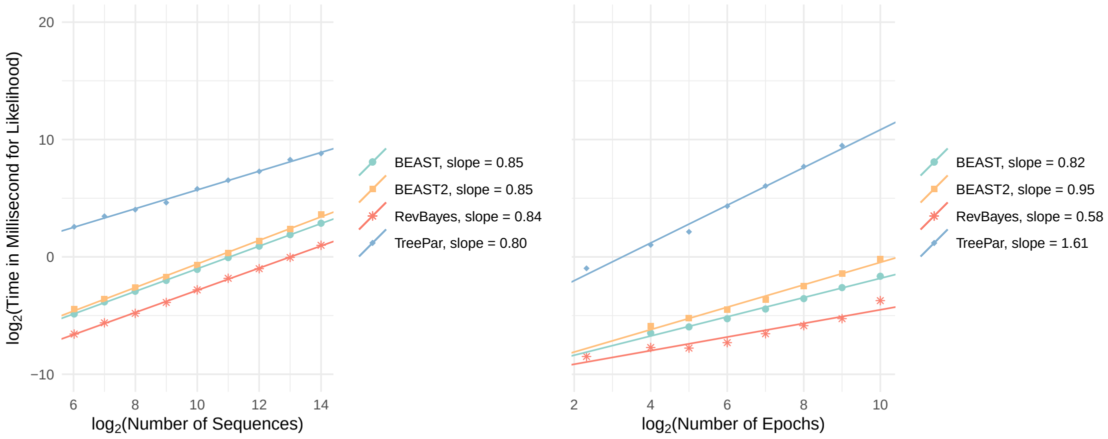

Figure D: Speed of implementations for the likelihood calculations of increasing number of sequences (left plot) or number of epochs (right plot) for EBDS model. Note the time and number of sequences/epochs are laid out according to a logarithmic scale with base 2.

In our analysis, we observe that for likelihood computations, the implementations in BEAST, BEAST2, and RevBayes offer similar speed performance when adjusting both the number of sequences and epochs. In contrast, the TreePar package consistently lags, being several hundred times slower than its counterparts across all tested scenarios. It is also the sole implementation that exhibits a quadratic scaling with the number of epochs. The algorithms of BEAST, BEAST2, and RevBayes seem to demonstrate approximately linear scaling relative to both tree size and model epochs. It's worth noting that RevBayes delivers the quickest calculation speed, which might be attributed to the inherent speed advantages of precompiled codes, particularly for quick likelihood calculations in our context. Result for TreePar with epochs exceeding than 512 is not not included as TreePar fail to process such large models.

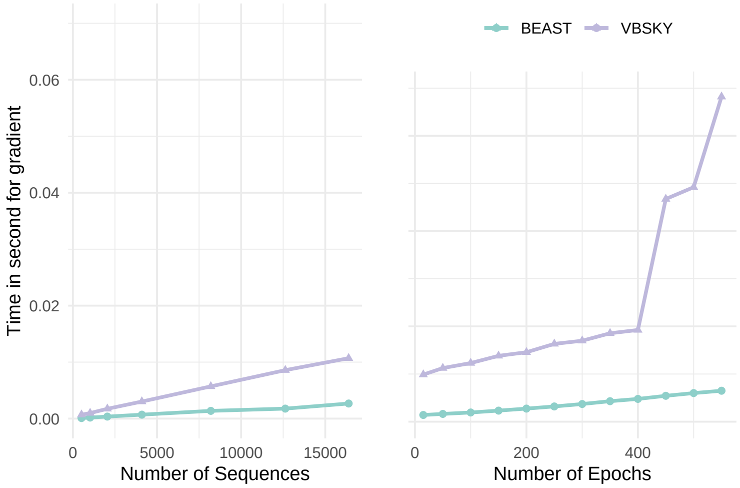

Figure E: Speed of implementations for and gradient calculations of increasing number of sequences (left plot) or number of epochs (right plot) for EBDS model.

In terms of gradient calculations, our analytical gradients deployed within BEAST is remarkably faster than VBSKY approach using automatic differentiation. The gradient computation scales approximately linearly with the number of sequences for both BEAST and VBSKY. However, while this linearity persists for BEAST wrt an increasing number of epochs, VBSKY shows a departure from linear scaling. Notably, the run time for the VBSKY method escalates from 0.02 seconds with 400 epochs to 0.04 seconds with 450 epochs. We further confirm that the runtime slowness exhibited in VBSKY is not due to memory issues or JIT compilation difficulty. However, without the ability to modify or closely examine the automatic differentiation library employed by VBSKY, identifying the specific causes of this non-linear scaling remains out of reach. Therefore, our analysis demonstrates that analytically calculating the gradients of the EBDS likelihood is critical for improving the running time of gradient based methods.

8 XML specification for the EBDS model using HMC sampler

BEAST data, likelihood, prior and sampling specification relies on extensive markup language (XML) elements. Comprehensive instructions for incorporating XML elements to-

gether to drive BEAST are provided in the How-to Guides on the BEAST community website https://beast.community/. In the instructions here, we address the construction and use of XML elements for the EBDS model and HMC transition kernels, which are the key components for the application of the results presented in this study.

The EBDS model XML elememt is specified as follows:

```
<newBirthDeathSerialSampling id="bdss" units="years" hasFinalSample="false"
    conditionOnSurvival="false">
    <birthRate>
        <parameter idref="bdss.birthRate"/>
    </birthRate>
    <deathRate gradientFlag = "false">
        <parameter idref="bdss.deathRate"/>
    </deathRate>
    <samplingRate>
        <parameter idref="bdss.samplingRate"/>
    </samplingRate>
    <samplingProbability gradientFlag = "false">
        <compoundParameter id="bdss.samplingProbability">
            <parameter id="samplingAtPresent" value="0" dimension="1" lower="0.0" upper=
                "1.0"/>
            <parameter id="otherSampling" value="0" dimension="DIM - 1" lower="0.0"
                upper="1.0"/>
        </compoundParameter>
    </samplingProbability>
    <treatmentProbability gradientFlag = "false">
        <parameter id="bdss.treatmentProbability" value="1.0" dimension="DIM" lower="0.0
            " upper="1.0"/>
    </treatmentProbability>
    <origin>
        <parameter id="bdss.origin" value="ORIGIN TIME" lower="0.0"/>
    </origin>
    <cutOff>
        <parameter value="CUT OFF TIME"/>
    </cutOff>
    <numGridPoints>
        <parameter value="DIM"/>
    </numGridPoints>
</newBirthDeathSerialSampling>
```

The option **conditionOnSurvival** controls whether the model conditions on the survival of at least one individual at the present time. Note that if we remove the compound parameter declarations, we need to set the initial parameter values for **birthRate**, **deathRate**, and **samplingRate** in this EBDS model block. Users can refer to the XML files for HIV examples in our provided Github repository to make the corresponding changes. For our analyses, we assume no intensive sampling events at the epoch switching times, so we set

the **samplingProbability** at other times to be 0. Users can modify this parameter to value between 0 and 1 to incorporate these intensive sampling events. We need to input a starting value for parameter **origin** which matches the length of the starting phylogeny or the fixed tree. The parameter **cutOff** governs the end point of our last epoch and **numGridPoints** specifies the number of epochs. The current setup of this XML block assumes equidistant epoch switching times. Users can modify the grid points by adding additional parameter **grids** in this EBDS model block. To illustrate, in our HIV example, we have:

```
<grids>
  <parameter value="0 3 6 8 10 12 14 16 18 25"/>
</grids>
```

Following the incorporation of XML elements for the selected prior distributions and models pertinent to joint phylogeny inference, we can shift our focus to the transition kernel or "operator" element. This block contains a unique operator, the **hamiltonianMonteCarloOperator**, differing from standard operators in that it requires a **jointGradient** object instead of a typical parameter object. BEAST internally retrieves the parameter from its gradient object. As we are dealing with gradients with respect to several different parameters, we can define **compoundGradient** elements as needed in advance. Specifically, we define the compound gradient using two new elements: the **gradient** element for thr priors and the **speciationLikelihoodGradient** element for EBDS model parameters. A sample HMC operator element is shown below.

The implementation of HMC necessitates user specification of two critical parameters: the step size **stepSize**, and the number of steps **nSteps**. Notably, BEAST features intrinsic auto-tuning capabilities, facilitating parameter tuning during active analysis. We can enable this measure by specifying **autoOptimize="true"** and declaring a value for **targetAcceptanceProbability**. We also include a **preconditioner** element here which is not mandantorily required for HMC. However, as highlighted in the main text, preconditioning the mass matrix based on the Hessian of the log-prior significantly improves the efficiency of our HMC sampler. Removing this element leads to a standard HMC transition kernel,

utilizing an identity matrix as the mass matrix. Finally, we can add in more operators in this element for other parameter of interests, that are not supported by HMC sampler.

```
<hamiltonianMonteCarloOperator weight="1" nSteps="15" stepSize="0.01" mode="vanilla"
drawVariance="1.0" autoOptimize="true" targetAcceptanceProbability="0.7"
preconditioningUpdateFrequency="3" preconditioningDelay="0">
  <jointGradient>
      <compoundGradient idref="grad.bdssIncrements.likelihood"/>
      <compoundGradient idref="grad.bdss.prior"/>
  </jointGradient>
  <preconditioner>
      <compoundPriorPreconditioner id="priorPreconditioner">
        <normalDistributionModel idref="birthRateAtPresentPriorDistribution"/>
        <bayesianBridgeDistribution idref="birthRateDeltaPrior"/>
        <normalDistributionModel idref="deathRateAtPresentPriorDistribution"/>
        <bayesianBridgeDistribution idref="deathRateDeltaPrior"/>
        <normalDistributionModel idref="samplingRateAtPresentPriorDistribution"/>
        <bayesianBridgeDistribution idref="samplingRateDeltaPrior"/>
      </compoundPriorPreconditioner>
  </preconditioner>
</hamiltonianMonteCarloOperator>
```

Supplementary References

1. Vasylyeva TI, Zarebski A, Smyrnov P, Williams LD, Korobchuk A, Liulchuk M, et al. Phylodynamics helps to evaluate the impact of an HIV prevention intervention. Viruses. 2020;12:469.

2. Centers for Disease Control and Prevention. Key Facts About Influenza (Flu);. Accessed: 2023-05-31. https://www.cdc.gov/flu/about/keyfacts.html.

3. Magee AF, Höhna S, Vasylyeva TI, Leaché AD, Minin VN. Locally adaptive Bayesian birth-death model successfully detects slow and rapid rate shifts. PLoS Computational Biology. 2020;16:e1007999.

4. Velásquez GE, Aibana O, Ling EJ, Diakite I, Mooring EQ, Murray MB. Time from infection to disease and infectiousness for Ebola virus disease, a systematic review. Clinical Infectious Diseases. 2015;61:1135-40.

5. Stadler T, Kühnert D, Bonhoeffer S, Drummond AJ. Birth–death skyline plot reveals

temporal changes of epidemic spread in HIV and hepatitis C virus (HCV). Proceedings of the National Academy of Sciences. 2013;110:228-33.

6. Bouckaert R, Vaughan TG, Barido-Sottani J, Duchêne S, Fourment M, Gavryushkina A, et al. BEAST 2.5: An advanced software platform for Bayesian evolutionary analysis. PLoS computational biology. 2019;15:e1006650.

7. R Core Team. R: A Language and Environment for Statistical Computing. Vienna, Austria; 2021. Available from: https://www.R-project.org/.

8. Höhna S, Landis MJ, Heath TA, Boussau B, Lartillot N, Moore BR, et al. RevBayes: Bayesian phylogenetic inference using graphical models and an interactive model-specification language. Systematic biology. 2016;65:726-36.

9. Ki C, Terhorst J. Variational phylodynamic inference using pandemic-scale data. Molecular Biology and Evolution. 2022;39:msac154.

10. Bradbury J, Frostig R, Hawkins P, Johnson MJ, Leary C, Maclaurin D, et al. JAX: Composable Transformations of Python+ NumPy Programs (v0. 2.5). Software available from https://github com/google/jax. 2018.
